# Primary Mucosal Melanoma: Clinical Experience from a Single Italian Center

**DOI:** 10.3390/curroncol31010042

**Published:** 2024-01-22

**Authors:** Rosa Falcone, Sofia Verkhovskaia, Francesca Romana Di Pietro, Giulia Poti, Tonia Samela, Maria Luigia Carbone, Maria Francesca Morelli, Albina Rita Zappalà, Zorika Christiana di Rocco, Roberto Morese, Gabriele Piesco, Paolo Marchetti, Cristina Maria Failla, Federica De Galitiis

**Affiliations:** 1 Department of Oncology, IDI-IRCCS, 00167 Rome, Italy; s.verkhovskaia@idi.it (S.V.); f.dipietro@idi.it (F.R.D.P.); g.poti@idi.it (G.P.); t.samela@idi.it (T.S.); m.morelli@idi.it (M.F.M.); a.zappala@idi.it (A.R.Z.); z.dirocco@idi.it (Z.C.d.R.); r.morese@idi.it (R.M.); g.piesco@idi.it (G.P.); paolo.marchetti@idi.it (P.M.); f.degalitiis@idi.it (F.D.G.); 2 Experimental Immunology Laboratory, IDI-IRCCS, 00167 Rome, Italy; marialuigia.carbone@idi.it (M.L.C.); c.failla@idi.it (C.M.F.)

**Keywords:** mucosal melanoma, immunotherapy, prognosis

## Abstract

(1) **Background:** Mucosal melanoma (MM) is a rare tumor, accounting for about 1% of all diagnosed melanomas. The etiology and pathogenesis of this tumor are unknown. It is characterized by an aggressive phenotype with poor prognosis and a low response rate to approved treatments. (2) **Methods:** We retrospectively analyzed the clinical features, treatments and outcomes of patients diagnosed with MM from different sub-sites (head and neck, gynecological and gastro-intestinal region) between 2013 and 2023 at our Institute. Survival times were estimated with the Kaplan–Meier method. Multivariate Cox regression was used to test the independence of significant factors in univariate analysis. (3) **Results:** Twenty-five patients were included in this study; the disease was equally distributed among females and males. The median age at diagnosis was 74 years old. The majority had MM originating from the head and neck (56%), particularly from the nasal cavity. BRAF V600 mutations were detected in 16% of the study population, limited to gastro-intestinal and gynecological MM. At diagnosis, at least half the patients (52%) had the disease located also at distant sites. The median overall survival (OS) in the whole study population was 22 months, with a longer OS for patients diagnosed at an early stage (38 months, *p* < 0.001). Longer OSs were reported for head and neck MM compared to other anatomic regions (0.06). Surgery of the primary tumor and radiotherapy were performed in 64% and 36% of the study population, respectively. Radiotherapy was performed only in head and neck MM. At multivariate analysis, the single factor that showed a reduced hazard ratio for death was radiotherapy. (4) **Conclusions:** The overall survival of MM from different sub-sites treated at our Italian Institution was 22 months, with better outcomes for early-stage disease and head and neck MM. Performing radiotherapy may have a protective effect on OS for head and neck MM. New treatment strategies are urgently needed to improve the outcome in this disease.

## 1. Introduction

Primary mucosal melanoma (MM) is a rare and aggressive tumor [1]. It arises from melanocytes distributed throughout mucosal membranes of the respiratory, gastrointestinal and genitourinary tract. Its prevalence varies according to ethnicity, being more common among the Asian population rather than Caucasians. Likely due to the hidden site of origin and the rich lymphatic and vascular environment, MM shows an aggressive phenotype with dismal prognosis [2]. Knowledge about pathogenesis, staging systems, appropriate treatments and predictive factors is lacking. Inaccuracies in staging the disease and the rarity of MM limit the inclusion in clinical trials and the chance to standardize treatments. Wide excision surgery is the primary treatment for localized disease [3]. Negative pathological margins are challenging due to the anatomic site of the primary tumor, with significant consequences on patient morbidity and with a high rate of local recurrence and distal metastases. Likewise, there is no unique staging system for MM [4]. For vaginal, urethral and anorectal melanoma, no TNM classification system is available. The addition of radiotherapy to surgical treatment of mucosal melanoma has been shown to improve locoregional tumor control [5], especially for head and neck MM, although the impact on overall survival (OS) is still debated. Despite the impressive progress in the treatment of cutaneous melanoma due to immune checkpoints inhibitors (ICIs) and target therapies, the efficacy of these treatments in MM remains limited compared to cutaneous disease. Trials dedicated to MM are lacking and only large studies in advanced melanoma have included a small number of patients with rare diseases (such as MM or occult melanoma). The efficacy of single- or dual-agent ICIs may also vary in different ethnic groups, with a lower tumor mutational burden (TMB) in the Chinese population as well as less frequent markers of pre-existing T-cell inflammation [6]. Unlike cutaneous melanoma, BRAF mutations are infrequent (8%) in MM [7]. Therefore, treatments with BRAF inhibitors rarely represent an option for these patients [8]. Some efforts have been made to study the whole-exome sequencing of MM without identifying additional mutated genes to target with drugs [7].

Few real-word experiences investigating outcomes and prognostic and predictive factors are available on MM, often with a limited sample size, focused on a particular anatomic region or on Asian individuals, not representative of Caucasian or other ethnic groups [9,10].

The objective of this study was to analyze clinical data and survival outcomes of MM patients diagnosed in our Institute in the last ten years.

## 2. Materials and Methods

Patients: All patients with confirmed histologic diagnosis of MM, evaluated at IDI-IRCCS from January 2013 to May 2023, were included in this retrospective study. In particular, demographic characteristics, tumor histology, stage, therapy and survival data were analyzed. For clinical and pathological staging, we distinguished between localized disease (to the tumor boundary), regional (with lymph node involvement or direct extension) and metastatic disease, as previously reported [11]. For head and neck MM, American Joint Committee on Cancer (AJCC) TNM Staging System 8th edition, 2017 was adopted [12]. This study was conducted according to the Good Clinical Practice Guidelines and the Declaration of Helsinki. This study was approved by the Institutional Review Boards of Istituto Dermopatico dell’Immacolata (IDI)-IRCCS. 

Statistical and epidemiological analysis: OS was defined as the time intervening from the date of diagnosis to the date of death. Event-free patients were censored at the date of the last follow-up. Progression-free survival (PFS) was defined as the time from the starting date of treatment to disease progression or death from any cause. Median time of survival was calculated and compared using the log-rank test. Multivariate Cox regression was used to test independence of significant factors in univariate analysis. *p* value < 0.05 was considered significant. The statistical analyses were performed using IBM SPSS Statistics 28.

## 3. Results

Over a period of 10 years, 25 patients with MM were evaluated at our Institute (Table 1). 

Men and women were equally affected. The median age at diagnosis was 74 years old. All, except one, were of Caucasian origin. The majority of MM originated from the head and neck (56%), in particular from the nasal cavity (40%). BRAF V600E mutations were present among 16% of MM; in particular, tumors harboring BRAF mutations came from the stomach (n = 1), anus (n = 2) and vulva (n = 1). N-RAS and c-KIT were available for a minority of patients. Among the 7 patients for whom N-RAS mutation analysis was performed, 2 (28%) had mutations. C-KIT mutation analysis was performed in six patients and a wild-type gene was detected in all of them. No family history for melanoma was reported among the 18 patients for whom data were available. The diagnosis was performed when the disease was localized to the primary site (with or without lymph node involvement) in 80% of MM with the remaining cases (20%) diagnosed as metastatic disease. Surgery of the primary tumor was performed in 64% of the study population, mostly for patients with localized disease. In one case, debulking surgery was arranged for advanced disease with the aim of controlling local symptoms. Surgery was not performed in those patients with metastatic disease (stage IVB head and neck MM, stage IV MM from other sites or locally advanced disease unsuitable for surgery, such as for gynecological MM). Surgery consisted of resection of the primary tumor in 13 patients, combined with lymph node resection in a further 3 patients. Margins were negative (R0) in 13 patients, positive in 2 patients and not estimable in 1 case for the fragmentation of the tumor. Radiotherapy was administered in one third (36%) of cases, limited to head and neck MM. 

Among the sub-group of head and neck MM (14 patients), 3 patients had stage II MM, 7 patients had stage III MM and 4 patients had stage IV MM. Resection of the primary tumor was feasible in 10 patients (71%). Among these 10 patients, neck dissection was performed in 3 patients because of suspicious lymph nodes. Surgery was performed for MM with a primary tumor smaller or equal to T4a (AJCC 8th edition), according to guidelines [12]. Postoperative radiotherapy was recommended and performed for all patients who underwent surgery, with a tumor larger or equal to pT3 N0. In addition, one patient received exclusive radiotherapy for cT4b N0 M0 MM; another one with stage II MM underwent radiotherapy for local relapse of the disease after surgery. Radiotherapy consisted of image-guided radiotherapy (IGRT) or intensity-modulated radiation therapy (IMRT) for almost the totality of patients (8/9 = 89%) with a dose ranging from 50 to 67.5 Gy (median 2 Gy/fraction). The other one received proton therapy by personal preference. 

Systemic treatment consisted of immunotherapy with ICIs as a single agent in 17 patients (71%): the majority of these patients (11/17, 65%) received anti-programmed cell death-1 (PD-1), and six patients received anti-cytotoxic T-lymphocyte antigen-4 (CTLA-4). BRAF inhibitor (BRAFi) plus MEK inhibitor (MEKi) were administered to four patients. Only two patients received chemotherapy (dacarbazine) because they were diagnosed before immunotherapy was available at our Institute. The median number of systemic treatments was 1 (range, 0–4). Eight patients (33%) received at least a second line of therapy. The median OS across the whole population study was 22 months, with a statistically significant difference among patients with localized, regional and metastatic disease (38 months, 9 months and 11 months, respectively; *p* < 0.010). OS was sensibly longer among MMs from the head and neck (26 months) compared to other anatomic sites (9 months, *p* = 0.06). 

At univariate analysis, staging, surgery and radiotherapy were statistically significant. Radiotherapy was confirmed to be an independent predictor of OS in a multivariate model including the three factors (Table 2). Limiting the analysis to head and neck MM, surgery and radiotherapy at univariate analysis were both significant. At multivariate analysis, RT was confirmed as an independent predictor of OS (*p* = 0.04). 

The median progression-free survival (PFS) to first-line therapy (PFS1) was 4 months, with an overall response rate of 22% (5/23) and a disease control rate of 48% (11/23). The median PFS to second-line therapy (PFS2) was 3 months. At the last follow-up, two patients (8%) were alive.

## 4. Discussion

This study evaluated 25 patients with MMs treated at a single Italian center over a period of 10 years. Due to the rarity of this disease in our country, few data are available on clinical features and outcomes of MMs diagnosed and treated in Italy. This is one of the largest series limited to a single site. Consistent with other studies on the Caucasian population, the head and neck was the privileged site for MM (56%), followed by the gastro-intestinal and gynecological tract [13,14]. The nasal cavity was the most common site of origin (40% of all MM). Both genders were equally represented with a slight prevalence of the female sex (52%). The diagnosis was performed on the elderly (median age, 74 years old). The lower percentage of females in our study population compared to other Caucasian patient cohorts may be justified by the low amount of genital MM [13,14]. BRAF V600 mutations were detected in 16% of MM, limited to gynecological and gastrointestinal MM. In the literature, BRAF mutations are reported in around 10% of MM [7], with a prevalence on the V600 codon (63%) and of 37% outside (non-V600). A previous investigation showed that vulvovaginal melanoma had a higher percentage of BRAF mutations (26%) compared to MM from other sites (8%), although the percentage of mutations in the V600 codon was low [15]. 

The median OS in the whole study population was 22 months, with a longer OS for patients diagnosed without metastases, particularly for those with localized disease at diagnosis (38 months). Metastatic stage at diagnosis is widely recognized as a prognostic factor in several studies [16,17], supporting the need of an early and prompt diagnosis of the disease. Age, gender and mutational status were found to have no effect on survival. In our analysis, patients with MM from the head and neck region showed a longer OS (although not statistically significant) compared to MM from other regions. In the literature, anatomic region is recognized as a prognostic factor for survival. Indeed, Al-Haseni et al., in the largest study of MM in a USA-based population, demonstrated a worse OS for gastrointestinal MM in comparison to head and neck and genitourinary melanoma [18]. Similar results were reported by Altieri et al. They showed that MM from less represented anatomic sites (spine, central nervous system, lung, pleura, liver, pancreas) conferred the worst prognosis [19]. 

Among the several factors tested at univariate analysis, radiotherapy was an independent predictor of OS, showing a significant protective effect. Although the role of radiotherapy in MM has not been evaluated in prospective trials, it is often recommended in the postoperative management of MMs at the primary site and neck dissection [12,20]. Most studies and metanalyses focus on radiotherapy in head and neck MM. In the largest one, adjuvant radiotherapy has been shown to confer a moderate survival advantage in head and neck MM compared to surgery alone with a reduced percentage of local recurrence [16,21,22]. More often, authors showed that adjuvant radiotherapy limited loco-regional recurrence, independently from OS [23,24]. Radiotherapy has been demonstrated to be a significant predictor of survival also in vulvar MM [25]. 

In the Western world, the activity of ICIs in MM has been demonstrated to be lower than in cutaneous melanoma (ORR 37% vs. 55–60%), with a similar safety profile [26]. A large analysis exploring the efficacy of ICIs in MM compared to cutaneous melanoma showed that nivolumab (anti-PD-1) combined with ipilimumab (anti-CTLA-4) had a greater efficacy than either agent alone for both diseases [26]. Patients with MM who received nivolumab alone and nivolumab combined with ipilimumab had a PFS of 3 months and 5.9 months, respectively. The ORR was 23.3% with nivolumab and 37.1% with the combo immune strategy. Similar results are reported in the literature with pembrolizumab, another anti-PD-1 antibody: the ORR to pembrolizumab was 19% and the median PFS was 2.8 months with a median duration of response (DOR) of 27.6 months and a median OS of 11.3 months [27]. Our results are in accordance with these data. Indeed, the median PFS to ICI in monotherapy, among our patients, was 4 months, with an overall response rate of 22% and a disease control rate of 48%. None of our patients received double ICIs. In a Chinese retrospective study with 162 patients, MM patients had a significantly longer PFS than those with cutaneous melanoma (*p* = 0.005), regardless of immunotherapy or chemotherapy [28]. The reason why MM is less responsive to ICI among Caucasians is still unknown. 

Understanding the role of the tumor microenvironment or of genetic variants on the response to ICIs is still in its infancy. No correlation has been found between the average tumor mutational burden (TMB) (6.23 mut/MB) and tumor response to ICIs [29]. The most frequent mutations in MM have been in SF3B1 (27%), KIT (18%) and NF1 (17%), a different pattern from cutaneous melanomas [30]. Moreover, there were genetic differences observed based upon the site of origin of the MM, with SF3B1 mutations being more frequent in MM of the anal/rectal area. The TP53 mutation was predominant in vulvovaginal melanoma [25,31]. Unfortunately, our analysis lacks information about these molecular data (TMB, genetic alterations) that may confirm or deny the previous findings in the Italian population. Indeed, some genetic signatures occur selectively in people coming from geographic regions where environmental or genetic factors may play a role in the development of MM [32]. 

Our study is a retrospective one and the interpretation of variables within this dataset is limited by the small sample size, the different stages of disease and the wide variety of treatments used (both in adjuvant and metastatic setting), across 10 years. Contrary to cutaneous melanoma or other types of cancer (such as head and neck, colorectal, breast or lung), there are no referral centers in Italy for MM. Very often, they are treated by specialists of the sub-sites (for instance, head and neck surgeons for head and neck MM, gynecologists for vulvovaginal MM) with a dispersion of data. Our analysis is the first attempt to combine data from MM from different sub-sites, treated at the same Institution, even if over a long period of time with the risk of losing some details for the retrospective nature of this work. Importantly, our analysis strengths the role of adjuvant radiotherapy as an independent predictor of survival, overcoming the benefit of reducing only the local recurrence rate. 

New treatment approaches, especially in the Asian population, are investigating the role of vascular endothelial growth factor receptor (VEGFR) inhibitors combined with ICIs, with promising results [33]. VEGF-A is strongly expressed in MMs, and in vivo studies showed that the inhibition of VEGF-A and PD-1 signaling suppresses tumor growth, increasing T-cell infiltration. Sheng et al. [34], in a phase IB trial limited to the Asian population, demonstrated that the combination of toripalimab (anti-PD-1) plus axitinib (a VEGF tyrosine kinase inhibitor) was well tolerated and had promising antitumor activity in patients with metastatic MM. Among 29 patients, the ORR was 48% with a median PFS of 7.5 months. Real world data among the largest population of Asians confirmed the activity of this combination, with an ORR of 30% and improved outcomes especially if therapy was used as a front-line [35]. Moreover, in a phase II study, even the combination of chemotherapy (carboplatin–paclitaxel) plus the VEGF-A inhibitor bevacizumab resulted in improved survival [36]. The median PFS reached in the chemo plus bevacizumab arm was 4.8 months, with an ORR of 20%. The median OS was 13.9 months. Although data were statistically significant compared to the control arm (chemotherapy alone), the results are not thrilling from a clinical point of view. In addition, these results need to be validated in randomized phase 3 trials and extended to non-Asian populations. 

New trials are investigating combined treatment strategies for MM. Table 3 shows the ongoing trials carried out all over the world, recruiting patients with resectable and locally advanced/metastatic MM. These studies exploit multimodality approaches based on combining immunotherapy with anti-angiogenic agents, chemotherapy or radiotherapy. Most trials are phase 2 trials and are being conducted in China or USA, with Italy and Europe hosting just a few studies not limited to MM. 

In conclusion, MM continues to be an aggressive disease with a poor prognosis and limited outcomes even when treated with multiple strategies (surgery, radiotherapy, chemotherapy and/or immunotherapy). Our study provides details on epidemiologic data of a rare disease, i.e., MM, at a referral center in Italy, supporting the benefit of radiotherapy. 

A few efforts have been made so far to better study this disease among the Italian population and to combine the efforts at a national level. A pilot study with a unique design and more arms should be encouraged to treat patients with MM, supporting the larger use of radiotherapy in the management of these patients. Genetic and microenvironmental analysis need to be integrated at the translational level, both in tissue or blood, to identify likely prognostic and predictive markers of the response to therapies. A computational approach may offer valid support to predict the response to treatments, identifying new drugs in such a rare disease.

## Figures and Tables

**Table 1:** Characteristics of the 25 subjects with MM.

Characteristic	MM n = 25
Gender	
female	13 (52%)
male	12 (48%)
Ethnicity	
Caucasian	24 (96%)
other	1 (4%)
Age at diagnosis (median, IQR)	74 (63–77)
Site of primary tumor	
nasal cavity	10 (40%)
ethmoid sinuses	1 (4%)
oral cavity	3 (12%)
esophagogastric	4 (16%)
anus	4 (16%)
gynecological	3 (12%)
BRAF status	
V600E	4 (16%)
WT	21 (84%)
Amelanotic	
Yes	2 (8%)
No/NA	23 (92%)
Staging	
Early	12 (48%)
Locally advanced	8 (32%)
Metastatic	5 (20%)
Surgery	
Yes	16 (64%)
No	9 (36%)
Radiotherapy	
Yes	9 (36%)
No	16 (64%)
Systemic therapy	
Yes	23 (92%)
ICIs	17
BRAFi+/−MEKi	4
CT	2
No	2 (8%)

WT: wild type; NA: not available; ICIs: immune checkpoint inhibitors; CT: chemotherapy; BRAFi: BRAF inhibitors; MEKi: MEK inhibitors.

**Table 2:** Exploratory analysis of effects of prognostic factors on overall survival.

Factor	Univariate		MULTIVARIATE	
	HR (95%, CI)	*p*	HR (95%, CI)	*p*
Age	1 (0.9–1.03)	0.5	–	-
Sex	0.9 (0.4–2)	0.8	–	-
BRAF status	1.07 (0.36–3.19)	0.9	–	-
Site of MM (H&N vs. others)	2.2 (1–5.2)	0.06	–	-
Staging	3.12 (1.62–6.02)	<0.001	2.40 (0.94–6.14)	0.07
Surgery	0.21 (0.07–0.61)	0.004	0.87 (0.19–3.96)	0.85
Radiotherapy	0.24 (0.09–0.64)	0.004	0.32 (0.11–0.92)	0.03
Systemic therapy (n°)	0.91 (0.61–1.36)	0.65	-	-

HR: hazard ratio; CI: confidence interval; H&N: head and neck; n°: number.

**Table 3:** Ongoing clinical trials on mucosal melanoma.

Clinical Trial Number—Title	Phase—Location	R	Setting	Experimental Arm	Control Arm	Primary Objective	Status
NCT04462965 Postoperative Adjuvant Treatment of Completely Resected Mucosal Melanoma Phase II Study	II—China	Y	Resected MM	Toripalimab + Temozolomide + Cisplatin	Placebo + Temozolomide + Cisplatin	RFS	Rec
NCT05111574 Using Nivolumab Alone or With Cabozantinib to Prevent Mucosal Melanoma Return After Surgery	II—USA, Canada	N	Resected MM	Nivolumab + Cabozantinib	Nivolumab	RFS	Rec
NCT04318717 Pembrolizumab and Hypofractionated Radiation Therapy for the Treatment of Mucosal Melanoma	II—USA	N	Resected MM	Pembrolizumab + RT	-	Local tumor control rate	Rec
NCT04879654 Toripalimab Combined with Radiotherapy and Chemotherapy in the Treatment of SNMM After Endoscopic Surgery (SNMM)	II—China	N	Resected SNMM	Toripalimab + RT + CT	-	OS	Rec
NCT04180995 Toripalimab in Combination with Axitinib in Patients With Localized Mucosal Melanoma	II—China	N	Neoadjuvant—being considered to be able to be completely resected	Toripalimab + Axitinib	-	pathological response (pCR + pPR) rate	Rec
NCT05545969 A Multicentre, Open Label, Phase II Study to Determine the Response to Neoadjuvant Pembrolizumab and Lenvatinib Followed by Adjuvant Treatment with Pembrolizumab and Lenvatinib in Mucosal Melanoma	II—Australia	N	Neoadjuvant—locally advanced MM	Pembrolizumab + Lenvatinib	-	pathological response (pCR + pPR) rate	NYR
NCT03313206 Neoadjuvant Treatment Associated With Maintenance Therapy by Anti-PD1 Immunotherapy in Patients With Resectable Head and Neck Mucosal Melanoma (IMMUQ)	II—France	N	Neoadjuvant—resectable head and neck MM	Pembrolizumab + Lenvatinib	-	DFS	Rec
NCT04622566 Lenvatinib and Pembrolizumab in Resectable Mucosal Melanoma	II—China	N	Neoadjuvant-Resectable MM	Lenvatinib + Pembrolizumab	-	pCR	NYR
NCT05384496 Phase 2 Study of Axitinib + PD-1 Blockadein Mucosal Melanoma With Pilot Addition of Stereotactic Body Radiotherapy or Ipilimumab in Select Progressors	II—USA	N	Advanced/ metastatic MM	Nivolumab + Axitinib	-	ORR	Rec
NCT05420324 A Study to Assess YH003 in Combination With Pembrolizumab and Albumin Paclitaxel Injection in Subjects With Unresectable/Metastatic Mucosal Melanoma	II—China	N	Advanced/ metastatic MM	YH003 + Pembrolizumab + Albumin Paclitaxel	-	ORR	Rec
NCT04830124 A Phase 2, Open Label, Multicenter, Cohort Study of Nemvaleukin Alfa (ALKS 4230) Monotherapy in Patients With Advanced Cutaneous Melanoma or Advanced Mucosal Melanoma Who Have Previously Received Anti-PD-[L]-1 Therapy—ARTISTRY-6	II—multiple sites (East-West World)	N	Advanced/ metastatic MM	Nemvaleukin Alfa	-	ORR	Rec
NCT05436990 Antitumor Activity of Vactosertib in Combination With Pembrolizumab in Acral and Mucosal Melanoma Patients Progressed From Prior Immune Check Point Inhibitor	II—Korea	N	Advanced/ metastatic MM	Vactosertib + Pembrolizumab	-	ORR	NYR
NCT05009446 Single Arm Study of Induction Chemoradiotherapy Combined With Surgery in the Treatment of Locally Advanced SNMM (SNMM)	I—China	N	Locally advanced SNMM	CT/RT + surgery	-	OS	Rec
NCT06041724 Envafolimab Combined With Recombinant Human Endostatin and First-line Chemotherapy Treat of Advanced Mucosal Melanoma	II—China	N	Advanced/ metastatic MM	Envafolimab + recombinant human endostatin + temozolomide + cisplatin	-	PFS	NYR
NCT05089370 Oral Decitabine/Cedazuridine (DEC-C) in Combination With Nivolumab for Patients With Mucosal Melanoma	I/II—USA	N	Advanced/ metastatic MM	Decitabine/Cedazuridine (DEC-C) + Nivolumab	-	RP2D of DEC-C	Rec

R: randomization; Y: yes; N: no; Rec: recruiting; NYR: not yet recruiting; ORR: objective response rate; USA: United States; RFS: recurrence-free survival; pCR: pathological complete response; pPR: pathological partial response; MM: mucosal melanoma; DFS: disease-free survival; SNMM: sinonasal mucosal melanoma; CT/RT: chemoradiotherapy; OS: overall survival; PFS: progression-free survival; RP2D: recommended phase 2 dose; RT: radiotherapy.

## Data Availability

The data presented in this study are available on request from the corresponding author.

## References

[1] Mihajlovic M., Vlajkovic S., Jovanovic P., Stefanovic V. (2012). Primary mucosal melanomas: A comprehensive review. Int. J. Clin. Exp. Pathol..

[2] Shuman A.G., Light E., Olsen S.H., Pynnonen M.A., Taylor J.M., Johnson T.M., Bradford C.R. (2011). Mucosal melanoma of the head and neck: Predictors of prognosis. Arch. Otolaryngol. Head Neck Surg..

[3] Seifried S., Haydu L.E., Quinn M.J., Scolyer R.A., Stretch J.R., Thompson J.F. (2015). Melanoma of the vulva and vagina: Principles of staging and their relevance to management based on a clinicopathologic analysis of 85 cases. Ann. Surg. Oncol..

[4] Cui C., Lian B., Zhang X., Wu D., Li K., Si L., Yang Y., Tian H., Zhou L., Chi Z. (2022). An Evidence-Based Staging System for Mucosal Melanoma: A Proposal. Ann. Surg. Oncol..

[5] Owens J.M., Roberts D.B., Myers J.N. (2003). The role of postoperative adjuvant radiation therapy in the treatment of mucosal melanomas of the head and neck region. Arch. Otolaryngol. Head Neck Surg..

[6] Shoushtari A.N., Bao R., Luke J.J. (2020). PD-1 Blockade in Chinese versus Western Patients with Melanoma. Clin. Cancer Res..

[7] Dumaz N., Jouenne F., Delyon J., Mourah S., Bensussan A., Lebbé C. (2019). Atypical. Cancers.

[8] Bai X., Mao L.L., Chi Z.H., Sheng X.N., Cui C.L., Kong Y., Dai J., Wang X., Li S.M., Tang B.X. (2017). BRAF inhibitors: Efficacious and tolerable in BRAF-mutant acral and mucosal melanoma. Neoplasma.

[9] Mao L., Qi Z., Zhang L., Guo J., Si L. (2021). Immunotherapy in Acral and Mucosal Melanoma: Current Status and Future Directions. Front. Immunol..

[10] Moya-Plana A., Herrera Gómez R.G., Rossoni C., Dercle L., Ammari S., Girault I., Roy S., Scoazec J.Y., Vagner S., Janot F. (2019). Evaluation of the efficacy of immunotherapy for non-resectable mucosal melanoma. Cancer Immunol. Immunother..

[11] Altieri L., Eguchi M., Peng D.H., Cockburn M. (2019). Predictors of mucosal melanoma survival in a population-based setting. J. Am. Acad. Dermatol..

[12] NCCN (2024). Head and Neck Cancers. NCCN Guidelines in Oncology.

[13] Heppt M.V., Roesch A., Weide B., Gutzmer R., Meier F., Loquai C., Kähler K.C., Gesierich A., Meissner M., von Bubnoff D. (2017). Prognostic factors and treatment outcomes in 444 patients with mucosal melanoma. Eur. J. Cancer.

[14] Sarac E., Amaral T., Keim U., Leiter U., Forschner A., Eigentler T.K., Garbe C. (2020). Prognostic factors in 161 patients with mucosal melanoma: A study of German Central Malignant Melanoma Registry. J. Eur. Acad. Dermatol. Venereol..

[15] Hou J.Y., Baptiste C., Hombalegowda R.B., Tergas A.I., Feldman R., Jones N.L., Chatterjee-Paer S., Bus-Kwolfski A., Wright J.D., Burke W.M. (2017). Vulvar and vaginal melanoma: A unique subclass of mucosal melanoma based on a comprehensive molecular analysis of 51 cases compared with 2253 cases of nongynecologic melanoma. Cancer.

[16] Aashiq M., Silverman D.A., Na’ara S., Takahashi H., Amit M. (2019). Radioiodine-Refractory Thyroid Cancer: Molecular Basis of Redifferentiation Therapies, Management, and Novel Therapies. Cancers.

[17] Cinotti E., Chevallier J., Labeille B., Cambazard F., Thomas L., Balme B., Leccia M.T., D’Incan M., Vercherin P., Douchet C. (2017). Mucosal melanoma: Clinical, histological and c-kit gene mutational profile of 86 French cases. J. Eur. Acad. Dermatol. Venereol..

[18] Al-Haseni A., Vrable A., Qureshi M.M., Mathews S., Pollock S., Truong M.T., Sahni D. (2019). Survival outcomes of mucosal melanoma in the USA. Future Oncol..

[19] Altieri L., Wong M.K., Peng D.H., Cockburn M. (2017). Mucosal melanomas in the racially diverse population of California. J. Am. Acad. Dermatol..

[20] Pittaka M., Kardamakis D., Spyropoulou D. (2016). Comparison of International Guidelines on Mucosal Melanoma of the Head and Neck: A Comprehensive Review of the Role of Radiation Therapy. In Vivo.

[21] Grant-Freemantle M.C., Lane O’Neill B., Clover A.J.P. (2021). The effectiveness of radiotherapy in the treatment of head and neck mucosal melanoma: Systematic review and meta-analysis. Head Neck.

[22] Li W., Yu Y., Wang H., Yan A., Jiang X. (2015). Evaluation of the prognostic impact of postoperative adjuvant radiotherapy on head and neck mucosal melanoma: A meta-analysis. BMC Cancer.

[23] Wushou A., Hou J., Zhao Y.J., Miao X.C. (2015). Postoperative adjuvant radiotherapy improves loco-regional recurrence of head and neck mucosal melanoma. J. Craniomaxillofac. Surg..

[24] Jarrom D., Paleri V., Kerawala C., Roques T., Bhide S., Newman L., Winter S.C. (2017). Mucosal melanoma of the upper airways tract mucosal melanoma: A systematic review with meta-analyses of treatment. Head Neck.

[25] Sinasac S.E., Petrella T.M., Rouzbahman M., Sade S., Ghazarian D., Vicus D. (2019). Melanoma of the Vulva and Vagina: Surgical Management and Outcomes Based on a Clinicopathologic Reviewof 68 Cases. J. Obstet. Gynaecol. Can..

[26] D’Angelo S.P., Larkin J., Sosman J.A., Lebbé C., Brady B., Neyns B., Schmidt H., Hassel J.C., Hodi F.S., Lorigan P. (2017). Efficacy and Safety of Nivolumab Alone or in Combination With Ipilimumab in Patients With Mucosal Melanoma: A Pooled Analysis. J. Clin. Oncol..

[27] Hamid O., Robert C., Ribas A., Hodi F.S., Walpole E., Daud A., Arance A.S., Brown E., Hoeller C., Mortier L. (2018). Antitumour activity of pembrolizumab in advanced mucosal melanoma: A post-hoc analysis of KEYNOTE-001, 002, 006. Br. J. Cancer.

[28] Zhang Y., Fu X., Qi Y., Gao Q. (2019). A study of the clinical characteristics and prognosis of advanced mucosal and cutaneous melanoma in a Chinese population. Immunotherapy.

[29] Buchbinder E.I., Weirather J.L., Manos M., Quattrochi B.J., Sholl L.M., Brennick R.C., Bowling P., Bailey N., Magarace L., Ott P.A. (2021). Characterization of genetics in patients with mucosal melanoma treated with immune checkpoint blockade. Cancer Med..

[30] Nassar K.W., Tan A.C. (2020). The mutational landscape of mucosal melanoma. Semin. Cancer Biol..

[31] Lorenz A., Kozłowski M., Lenkiewicz S., Kwiatkowski S., Cymbaluk-Płoska A. (2022). Cutaneous Melanoma versus Vulvovaginal Melanoma-Risk Factors, Pathogenesis and Comparison of Immunotherapy Efficacy. Cancers.

[32] Newell F., Kong Y., Wilmott J.S., Johansson P.A., Ferguson P.M., Cui C., Li Z., Kazakoff S.H., Burke H., Dodds T.J. (2019). Whole-genome landscape of mucosal melanoma reveals diverse drivers and therapeutic targets. Nat. Commun..

[33] Li S., Wu X., Yan X., Zhou L., Chi Z., Si L., Cui C., Tang B., Mao L., Lian B. (2022). Toripalimab plus axitinib in patients with metastatic mucosal melanoma: 3-year survival update and biomarker analysis. J. Immunother. Cancer.

[34] Sheng X., Yan X., Chi Z., Si L., Cui C., Tang B., Li S., Mao L., Lian B., Wang X. (2019). Axitinib in Combination With Toripalimab, a Humanized Immunoglobulin G. J. Clin. Oncol..

[35] Tang B., Mo J., Yan X., Duan R., Chi Z., Cui C., Si L., Kong Y., Mao L., Li S. (2021). Real-world efficacy and safety of axitinib in combination with anti-programmed cell death-1 antibody for advanced mucosal melanoma. Eur. J. Cancer.

[36] Yan X., Sheng X., Chi Z., Si L., Cui C., Kong Y., Tang B., Mao L., Wang X., Lian B. (2021). Randomized Phase II Study of Bevacizumab in Combination With Carboplatin Plus Paclitaxel in Patients With Previously Untreated Advanced Mucosal Melanoma. J. Clin. Oncol..

